# A molecular phylogeny of Porcellionidae (Isopoda, Oniscidea) reveals inconsistencies with present taxonomy

**DOI:** 10.3897/zookeys.801.23566

**Published:** 2018-12-03

**Authors:** Andreas C. Dimitriou, Stefano Taiti, Helmut Schmalfuss, Spyros Sfenthourakis

**Affiliations:** 1 Department of Biological Sciences, University of Cyprus, Panepistimiou Ave. 1, 2109 Aglantzia, Nicosia, Cyprus University of Cyprus Nicosia Cyprus; 2 Istituto di Ricerca sugli Ecosistemi Terrestri, Consiglio Nazionale delle Ricerche, Via Madonna del Piano 10, 50019 Sesto Fiorentino (Florence), Italy Istituto di Ricerca sugli Ecosistemi Terrestri, Consiglio Nazionale delle Ricerche Florence Italy; 3 Museo di Storia Naturale dell’Università di Firenze, Sezione di Zoologia “ La Specola”, Via Romana 17, 50125 Florence, Italy Museo di Storia Naturale dell’Università di Firenze Florence Italy; 4 Staatliches Museum für Naturkunde, Stuttgart, Rosenstein 1, 70191 Stuttgart, Germany Staatliches Museum für Naturkunde Stuttgart Germany

**Keywords:** Crinocheta, genetic markers, monophyly, node dating, taxonomic revision, woodlice

## Abstract

Porcellionidae is one of the richest families of Oniscidea globally distributed but we still lack a comprehensive and robust phylogeny of the taxa that are assigned to it. Employing five genetic markers (two mitochondrial and three nuclear) we inferred phylogenetic relationships among the majority of Porcellionidae genera. Phylogenetic analyses conducted via Maximum Likelihood and Bayesian Inference resulted in similar tree topologies. The mtDNA genes cytochrome oxidase I (COI) and 16s rRNA (16s) were used for clade dating using previously published mutation rates. Our results provide evidence against the monophyly of both Porcellionidae and the largest genus of the family *Porcellio*. These results are compared to previous published work based on morphological evidence. The genera *Leptotrichus* and *Brevurus* are not grouped with the rest of Porcellionidae whereas Agnaridae are grouped with part of Porcellionidae. *Armadillidium* and *Schizidium* (Armadillidiidae) occupy a basal position on the phylogenetic tree. Even though the African genera *Tura* and *Uramba* (distributed in East Africa) are grouped together there is no general geographical pattern in other sub-clades. Additional taxonomic issues that arise in this work such as the assignment of the recently described genus *Levantoniscus*, arealso discussed. The status of Porcellionidae should be further revised and morphological characters traditionally used in Oniscidea taxonomy should be reconsidered in view of molecular evidence. The origin of the monophyletic clade within Porcellionidae as indicated in the present work is dated back to the Oligocene (~32 mya).

## Introduction

The Oniscidea family Porcellionidae is one of the richest in species, with 333 species, belonging to 19 genera, currently assigned to it (Sfenthourakis and Taiti 2015). Family members are unable to conglobate, with the exception of the genus *Atlantidium* Arcangeli, 1936. There is remarkable morphological variation among Porcellionidae species and genera, especially in head structure, pleotelson, and body shape. Familial assignment of taxa is based mostly on the combination of two character states, namely an antennal flagellum with two articles and the presence of monospiracular, covered lungs on the first two pairs of pleopods (Schmidt 2003). However, certain authors, based on morphological and recent molecular work, suggest that these characters could be symplesiomorphies, as they are not exclusively found in Porcellionidae (Schmalfuss and Ferrara 1978, Schmidt 2003).

Different authors have found Porcellionidae to be closely related with Oniscidae, Trachelipodidae, Cylisticidae, Agnaridae or Armadillidiidae (Michel-Salzat and Bouchon 2000, Mattern 2003, Schmidt 2008, Lins et al. 2017). Furthermore, monophyly of the most species-rich genera, *Porcellio* Latreille, 1804 and *Porcellionides* Miers, 1877, has been debated on the basis of both morphology (Vandel 1962, Schmalfuss 1992, 1998) and molecular evidence (Michel-Salzat and Bouchon 2000, Mattern 2003). More specifically, some *Porcellionides* species appear to be more closely related to the genus *Porcellio* (Michel-Salzat and Bouchon 2000, Mattern 2003) or even to the genus *Cylisticus* Schnitzler, 1853 that belongs to another family (Cylisticidae),than to other congeneric species (Michel-Salzat and Bouchon 2000). Hence, also the monophyly of the family has been repeatedly questioned on the basis of both morphological and genetic data (Schmalfuss 1989, Michel-Salzat and Bouchon 2000, Mattern and Schlegel 2001, Schmidt 2003, 2008).

Members of Porcellionidae were originally reported from the circum-Mediterranean region, Atlantic islands, Arabian Peninsula and East Africa. Nowadays they are known from all over the world, being introduced into many regions by human activities (Schmidt 2003). Porcellionidae are considered to be among the isopod species that are better adapted to terrestrial environments, and they can be found in a wide range of habitats, from tropical rainforests to deserts (Schmidt 2003, Medini-Bouaziz et al. 2017).

The present study aims to a more detailed investigation of phylogenetic relationships among genera of Porcellionidae, using two mitochondrial and three nuclear genes that allow estimation of divergence times among extant taxa.

## Materials and methods

### Sampling

Isopod specimens belonging to five Porcellionidae genera, one to Trachelipodidae (*Levantoniscus* Cardoso, Taiti & Sfenthourakis, 2015) and two to Armadillidiidae (*Armadillidium* Brandt, 1831 and *Schizidium* Verhoeff, 1901) were collected on Cyprus between 2014 and 2016. Additional specimens came from the collection of the Istituto per lo Studio degli Ecosistemi, deposited in the Museum of Natural History of the University of Florence, and from the personal collection of one of the authors (H.S.). Members of the families Armadillidiidae, Agnaridae and Trachelipodidae that are assumed to be closely related to Porcellionidae were included in the analyses to test the monophyly of the latter, whilst specimens of the more distant families Scyphacidae (*Actaeciaeuchroa* Dana, 1853) and Philosciidae (*Chaetophilosciaelongata* (Dollfus, 1884) were included as outgroups. More details about specimens used are given in Table 1.

We were not able to include specimens of five Porcellionidae genera, namely the monotypic *Congocellio* Arcangeli, 1950 and *Tropicocellio* Arcangeli, 1950, both distributed in the Democratic Republic of the Congo, *Dorypoditius* Verhoeff, 1942 from Mozambique, *Atlantidium* Arcangeli, 1936 form Madeira, and *Pondo* Barnard, 1937 from South Africa (Pondoland and Natal).

**Table 1.** Species, locality of origin, available sequence data from targeted genes, and GenBank accession numbers of individuals used in the molecular phylogenetic analyses.

Species *(code)*	*Locality*	*Genes*					Acc. No
		COI	16s	18s	28s	NAK	
** Porcellionidae **							
*Proporcelliovulcanius* (Verhoeff, 1908) (1)	Cyprus (Larnaca)	√	√		√	√	MG887933/MG887948/-/MG887988/MG887906
*Agabiformiusexcavatus* Verhoeff, 1941 (2)	Cyprus (Paphos)		√	√	√	√	-/MG887955/MG887969/ MG888009/MG887921
*A.excavatus* (3)	Cyprus (Paphos)		√			√	-/MG887956/-/-/MG887922
*Porcelliolaevis* Latreille, 1804 (4)	Cyprus (Lemesos)	√	√	√	√	√	MG887936/MG887957/MG887986/MG887993/MG887913
*P.laevis* (5)	Cyprus (Lemesos)	√	√	√	√	√	MG887937/MG887958/MG887987/ MG887994/MG887914
*Porcellionidespruinosus* (Brandt, 1833) (6)	Cyprus (Larnaca)	√	√		√	√	MG887934/MG887949/-/MG888010/MG887907
*P.pruinosus* (7)	Cyprus (Larnaca)	√	√		√	√	MG887935/ MG887950/-/ MG887989/MG887908
*Leptotrichuskosswigi* Strouhal, 1960 (8)	Cyprus (Paphos)				√	√	-/-/-/MG888013/MG887915
*L.kosswigi* (9)	Cyprus (Paphos)		√	√	√	√	-/MG887963/MG887970/ MG888014/MG887916
*Porcellionasutus* Strouhal, 1936 (10)	Greece (Parnon)	√	√		√	√	MG887944/ MG887953/-/MG887998/MG887910
*P.nasutus* (11)	Greece (Parnon)		√	√	√	√	-/MG887954/MG887980/ MG887999/MG887911
*Tura* sp. (12)	Kenya (Mombasa)	√	√	√	√	√	MG887946/ MG887966/ MG887983/MG888001/MG887920
*Caeroplastesporphyrivagus* (Verhoeff, 1918) (13)	France (Toulon)	√		√	√		MG887932/-/ MG887981/ MG887990/ -
*Urambatriangulifera* Budde-Lund, 1910 (14)	Kenya (Aberdare National Park)		√		√	√	-/ MG887961/-/MG888002/MG887923
*Thermocellio* sp. (15)	Tanzania (Dar es Salaam)		√		√		-/ MG887962/-/ MG887995/-
*Lucasiuspallidus* (Budde-Lund, 1885) (16)	Italy (Sardinia)			√	√	√	-/-/MG887974/ MG887992/MG887917
*Micatardus* (Budde-Lund, 1885) (17)	Italy (Sardinia)		√		√		-/ MG887959/-/MG887996/-
*Acaeroplastesmelanurusmelanurus* (Budde-Lund, 1885) (18)	Italy (Sardinia)	√	√	√	√	√	MG887945/ G887960/MG887982/ MG887991/MG887912
*Soteriscuslaouensis* Taiti & Rossano, 2015 (19)	Morocco (Tirinesse)	√	√	√	√	√	MG887931/MG887964/MG887975/MG887997/MG887918
*Brevurusmasandaranus* Schmalfuss, 1986 (20)	Iran				√	√	-/-/-/MG888008/MG887919
*Porcellionidescilicius* (Verhoeff, 1918) (21)	Cyprus (Nicosia)					√	-/-/-/-/MG887909
** Trachelipodidae **							
*Levantoniscusbicostulatus* Cardoso, Taiti & Sfenthourakis, 2015 (22)	Cyprus (Paphos)			√	√	√	-/-/MG887976 /MG888000/MG887928
*Trachelipusaegaeus* (Verhoeff, 1907) (26)	Greece (Naxos)	√	√	√		√	EF659961/KF891440/ MG887984 /-/MG887925
** Agnaridae **							
*Hemilepistusklugii* (Brandt, 1933 (23)	Iran (Isfahan)	√	√	√	√	√	MG887938/MG887951/MG887978 /MG888011/MG887926
*H.schirazi* Lincoln, 1970 (24)	Iran (Shahreza)	√	√	√	√	√	MG887939/MG887952/MG887979 /MG888012/MG887927
*Agnaramadagascariensis* (Budde-Lund, 1885) (25)	U.A.E.			√	√	√	-/-/MG887977 /MG888003/MG887924
** Armadillidiidae **							
*Armadillidiumvulgare* (Latrteille, 1904) (27)	Cyprus (Limassol)	√	√	√	√		KR424609/AJ419997/ MG887972/MG888006/-
*Schizidiumfissum* (Budde-Lund, 1885) (28)	Cyprus (Paphos)			√	√		-/-/MG887973/MG888005/-
** Philosciidae **							
*Chaetophilosciaelongata* (Dollfus, 1884) (29)	Italy (Sardinia)	√	√	√	√	√	KJ668161/AJ388091/MG887971/MG888004/-/MG887929
** Scyphacidae **							
*Actaeciaeuchroa* Dana, 1853 (30)	New Zealand	√	√	√	√	√	GQ302701/AJ388093/MG887985/MG888007/MG887930

### Molecular analyses

Fresh specimens were placed in 96% alcohol immediately after collection and stored at -20 °C. The majority of samples from museums and private collections had been preserved in 70% alcohol. Whole animals or legs of larger specimens were used for extraction of total genomic DNA using DNeasy Blood and Tissue Kit (Qiagen, Hilden, Germany) following manufacturer’s instructions. NanoDrop 2000/200c (Thermo Fisher Scientific Inc., USA) was used to determine the final concentration and purity (A260/A280nm absorption rate) of DNA extractions.

### DNA extraction amplification and sequencing

The following mitochondrial and nuclear genetic loci were targeted using common PCR procedures: partial mitochondrial cytochrome *c* oxidase subunit 1 (COI), ribosomal 16S rRNA (16s), the nuclear, non-coding 18S ribosomal RNA (18s) and 28S ribosomal RNA (28s), and the protein coding Sodium-Potassium Pump (NAK). Mitochondrial COI and 16s genes were successfully amplified using the universal LCO1490/HCO2198 (Folmer et al. 1994) and the widely used 16sar/16sbr and 16sar-intsf (Palumbi 1996, Parmakelis et al. 2008) primers, respectively. The primer pairs 18sai/18sbi and 18Aimod/700R (Hermann and Wägele 2001, Raupach et al. 2009) were used for the amplification of 18s, and the 28sa/28sb pair (Whiting et al. 1997) was used successfully for all available samples. Finally, the protein coding NAK amplicons were targeted with NAK for-b/NAK rev 2 (Tsang et al. 2008) and the newly designed reverse primer NAK 638R: 5’-GGD RGR TCR ATC ATD GAC AT -3’.

All PCR reactions were performed in a Veriti thermal cycler (Applied Biosystems, USA) with the following common steps: a) initial denaturation for 5 min at 94 °C, followed by b) 5 cycles of 3 minutes equally separated at 94 °C/60 °C/72 °C, c) 5 cycles of 3 minutes equally separated at 94 °C/55 °C/72 °C, d) 10 cycles of 3 minutes equally separated at 94 °C/50 °C/72 °C, e) 10 cycles of 3 minutes equally separated at 94 °C/47 °C/72 °C, f) 10 cycles of 3 minutes equally separated at 94 °C/42 °C/72 °C, and g) a final extension step of 72 °C for 10 min. Beyond fresh specimens, this touchdown PCR approach with 50 cycles in total allowed us to successfully amplify genes from ill-preserved samples increasing specificity, sensitivity and yield, eliminating aspecific products (Korbie and Mattick 2008).

The final reaction volume in all cases was 20 μL, and consisted of 0.1 µL of Kapa *Taq* DNA Polymerase (5U/μL), 1.2 μL of 25 mM MgCl_2_, 2 μL of Kapa PCR buffer A, 0.6 μL of 10 mM dNTP (Kapa) 0.6 μL of each primer (10 µM) and >10 ng of DNA template. PCR product purification was made using Qiaquick Purification Kit (Qiagen, Germany) under manufactures protocol instructions. Both DNA strands of purified products were sequenced at Macrogen facilities (Amsterdam, The Netherlands).

### Alignments and genetic divergence

Sequence chromatograms were manually edited and assembled with CodonCode Aligner (v. 3.7.1; CodonCode Corp., USA). Separate multiple alignments for each gene/data set were performed using MAFFT v.7 (Katoh et al. 2002). Our data were further enriched by a limited number of publicly available NCBI GenBank mtDNA sequences (Table 1). The final concatenated data set was partitioned by gene into five distinct data blocks. The optimal nucleotide substitution models were identified using PartitionFinder v.1.1.1 (Lanfear et al. 2012). Three independent runs in PartitionFinder were applied, using the greedy search algorithm with linked branch lengths in calculations of likelihood scores under the Bayesian Information Criterion (BIC). The difference between these three runs was the restriction of candidate models to only those that are implemented in MRBAYES v.3.2.6 (Ronquist et al. 2012), BEAST v. 2.3.0 (Bouckaert et al. 2014) or RAxML v. 8.1.21 (Stamatakis 2014). Models that included both gamma distribution and invariable sites were neglected (Yang 2006).

### Phylogenetic analyses

Construction of phylogenetic trees was conducted using Bayesian inference (BI) and Maximum Likelihood (ML) methods. The analysis of BI was implemented in MRBAYES v. 3.2.6 (Ronquist et al. 2012) with four independent runs and eight chains per run for 3 × 10^7^ generations, with a sampling frequency of 100. Consequently, the summaries of BI were based on 3 × 10^5^ sampled trees from each run. The convergence and stationarity of each run was evaluated by monitoring the average standard deviation of split frequencies of the four simultaneous and independent runs in MRBAYES, and further by inspection of generation versus log probability of the data plot viewed in TRACER v.1.5.0 (Rambaut and Drummond 2007). The -ln value reached stationarity well before pre-requested 10^7^ generations. From the sampled trees, 25% were discarded as burn-in phase. Therefore a majority rule consensus tree relied on 300,004 trees and posterior probabilities were calculated as the percentage of samples recovering any particular clade (Huelsenbeck and Ronquist 2001).

RAxML (v. 8.1.21) (Stamatakis 2014) was recruited for Maximum Likelihood analyses which were conducted using the RAxMLGUI v.1.5 platform (Silvestro and Michalak 2012). The GTR+G model of evolution was used for the estimation of parameters for each partition. The optimum ML tree was selected after 500 iterations and the reliability of the branches was assessed by 1,000 thorough bootstrap replicates (Felsenstein 1985).

### Clock calibration and divergence time estimation

Molecular dating of clades was inferred using BEAST v. 2.3.0 (Bouckaert et al. 2014). The appropriate model of nucleotide substitution, as indicated by PartitionFinder under the BIC criterion was implemented for each marker in our partitioned analysis. Due to the absence of reliable geological or fossil data related to taxa included in our analyses, time of divergence was calibrated based on available gene-specific substitution rates. More specifically, the substitution rates of the mitochondrial genes 16s and COI were used as reported from previous studies for isopods (Held 2001, Poulakakis and Sfenthourakis 2008, Kamilari et al. 2014). Clock rate was set at 0.0007 (substitutions per site per Myr) for 16s and 0.0082 (min rate 0.0078, max 0.0086) for COI.

Four independent runs were performed for 100 million generations, each sampling every 5,000^th^ generation. An uncorrelated lognormal relaxed clock under a Yule tree prior and the default options for all other prior and operator settings, were used in each case. Trace plots were inspected in order to compare the divergence estimates across runs and ensure the convergence of Markov Chain Monte Carlo chains using TRACER v. 1.5 (Rambaut and Drummond 2007). Resulting log files were combined, after removing 10% as burn-in, using LOGCOMBINER v.2.3.0 (Bouckaert et al. 2014). A maximum clade credibility tree exhibiting the means of node heights was constructed with TREEANNOTATOR v.2.3.0 (Bouckaert et al. 2014).

## Results

At least four out of five targeted genes were successfully amplified and sequenced for the great majority of available individuals, with final DNA extraction yield over 20 ng/μl and A260/A280 purity rate over 1.5.Since some important samples were old (collected more than two decades ago, mainly from Africa) or ill-preserved for a long time (i.e., in 70% alcohol) we didn’t manage to retrieve sequences from all targeted genes. However, specimens not represented by all gene fragments were also included in the analyses. The final concatenated alignment obtained consisted of 3,841 base pairs (bp). More details about the aligned sequences length, conserved, variable and parsimony-informative sites for each gene are given in Table 2.

**Table 2.** Aligned bases length, conserved, variable, and parsimony-informative sites for each gene used in the present analysis.

Gene	Alignment length (bp)	Conserved sites	Variable sites	Parsimony informative sites
**COI**	655	214	434	302
**16S**	454	151	277	211
**18S**	863	417	332	177
**28S**	1167	314	827	567
**NAK**	702	512	188	109

Available sequences were separated in different groups at the genus level except for *Porcellio* species which were treated as different groups due to the alleged non-monophyly of the genus. Between groups p-genetic distances for each gene are given in Suppl. material 1.

The best-fit nucleotide substitution models for each partition/gene selected under the BIC criterion were (for both MRBAYES and BEAST) the HKY+G+X, HKY+G+X, TRNEF+G, TRN+G, TRN+G+X and GTR+G+X for COI, 16s, 18s, 28s and NAK genes, respectively. The selected model under --raxml commandline option at PartionFinder was the GTR+G (-ln =26511.0556641) for all genes.

**Figure 1.**
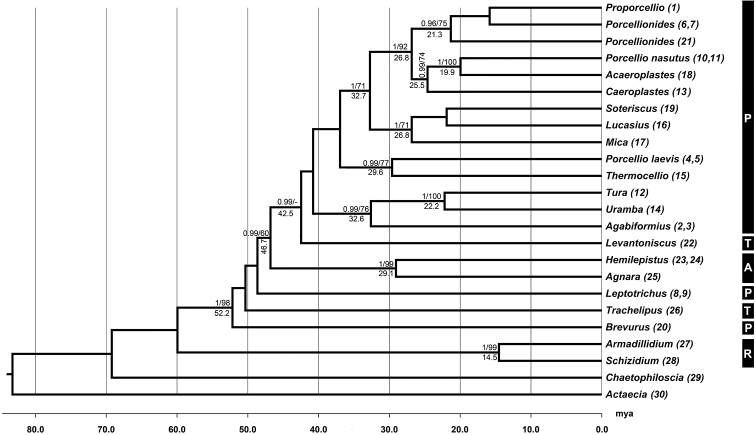
Dated phylogram based on concatenated data set including five genes (COI, 16s, 18s, 28s, NAK), generated using a relaxed lognormal clock in BEAST. BI posterior probabilities (>0.9) and ML bootstrap values (>60) are presented above the nodes. Estimated mean divergence time is given below the nodes only where nodes are statistically supported or the topology was identical between BI, ML and BEAST analyses. Subclades including individuals from more than one species have been collapsed to genus level, since all (except *Porcellio*) were monophyletic. Abbreviations: P. Porcellionidae, T. Trachelipodidae, A. Agnaridae, R. Armadillidiidae. Numbers in parentheses after each taxon name refer to numbering of taxa in Table 1.

Maximum Likelihood and Bayesian Inference analyses (implemented both in BEAST and MRBAYES) resulted into phylogenetic trees with similar, well-supported topologies. Given the congruence among the results of the two methods, only the Bayesian tree is presented herein (Figure 1). The ML tree is given in Suppl. material 1: (Figure S1). The separate analysis of different gene markers showed that the concatenated tree topology is mainly determined by nuclear genes. Missing data, and possibly also the depth of the phylogeny, led to largely unresolved trees for mtDNA markers. Nevertheless, these were used mainly to estimate node dates based on published mutation rates. The poor mtDNA-based resolution did not affect the final tree, given that the tree based solely on nuclear genes (see Suppl. material 1) has identical topology.

Our results provide evidence against the monophyly of both the family Porcellionidae and the genus *Porcellio*. *Brevurus* appears to belong to a supported distant clade, external to that formed by the remaining Porcellionidae+Trachelipodidae+Agnaridae. *Leptotrichus* is an external branch to Agnaridae + part of Porcellionidae. Monophyly of Agnaridae is supported. *Levantoniscus* forms the sister clade of all monophyletic Porcellionidae. Finally Armadillidiidae branches early in the tree, not showing any close relationship to Porcellionidae.

The African genera *Tura* and *Uramba* are sister taxa sharing a common ancestor at around 22.2 mya (95% HPD 12.1 – 33.5 mya) and are grouped with *Agabiformius*. On the other hand, *Thermocellio*, also distributed in Kenya and the neighboring Tanzania, appears to be more closely related to *Porcelliolaevis*, native to Europe and North Africa. Another African/Atlantic genus, *Soteriscus*, forms a well-supported clade with *Lucasius* and *Mica* that are distributed in Africa and on some Mediterranean islands. The Mediterranean genera *Acaeroplastes*, *Caeroplastes*, *Porcellionides* and *Proporcellio*, together with part of *Porcellio*, are grouped in the most derived clade that diverged at around 27 mya.

The genus *Porcellio* as currently perceived is represented in two well-supported separate clades. *P.laevis* groups with *Thermocellio* while *P.nasutus* with *Acaeroplastes* in a clade also including *Caeroplastes*.

Genetic distances between Porcellionidae genera (or species in the case of the non-monophyletic *Porcellio*) varied significantly among genes. The range of variation per gene is: COI: 16.9–50.3 %; 16s: 16.9–36.5 %; 18s:3.6–28.5 %; 28s:0.4–44.2%; NAK: 2.3–9.1%. The p-distances between *Trachelipus* and *Agnara* for NAK, and *P.laevis* and *Lucasius* for 18s, could be artifacts due to the comparatively shorter sequence length in *Agnara* and *P.laevis*, respectively (see Suppl. material 1).

It is worth noticing also that minimum and maximum distances are not exhibited by the same taxa for all genes. More specifically, highest / lowest genetic divergence is found between the following groups: *Tura* - *Porcellionasutus* / *Soteriscus* – *Leptotrichus* (16s), *Porcelliolaevis* – *Lucasius* / *Proporcellio* - *Porcellionides* (COI), *Agabiformius* - *Porcellionasutus* / *Caeroplastes* - *Acaeroplastes* (18s), *Brevurus* - *Thermocellio* / *Porcelliolaevis* - *Thermocelio* (28s) and *Uramba* – *Brevurus* / *Proporcellio* - *Porcellionides* (NAK). The allegedly congeneric *Porcellio* species never exhibit a minimum genetic distance.

## Discussion

This is the first comprehensive study aiming to resolve phylogenetic relationships among Porcellionidae genera using a multi-locus approach, thus increasing reliability of results. Our findings undermine the monophyly of both the family Porcellionidae and the genus *Porcellio*, in line with suggestions by previous authors (Schmalfuss 1989, Mattern 2003, Michel-Salzat and Bouchon 2000, Schmidt 2003, 2008).

The extremely high genetic distances, which reached up to 50.3 in mtDNA and 44.2 in nDNA, are confirming the vast divergence among taxa within Porcellionidae. Observed inconsistencies of group distances among different genes highlight the usefulness of the multi-locus approach followed herein for a reliable phylogenetic reconstruction of the taxa examined.

In view of the herein estimated phylogeny, a monophyletic Porcellionidae should exclude *Brevurus* and *Leptotrichus*. Moreover, the supposedly subtle morphological differences between *Leptotrichus* and *Agabiformius* that had led to a presumed sister-group relationship between these genera, are misleading, since they are found to be very distant (Schmalfuss 2000, Verhoeff 1908). *Brevurus* has been proposed as a possible synonym of *Porcellium* Dahl, 1916 (a genus of Trachelipodidae) (Khisametidonova and Schmalfuss 2012), an hypothesis that cannot be evaluated in view of our results.

The genus *Levantoniscus*, tentatively assigned to Trachelipodidae (Cardoso et al. 2015), has been found to be closer to the monophyletic subgroup of Porcellionidae. Given that the genus appears as the sister clade of all remaining monophyletic Porcellionidae, we cannot propose the assignment of this taxon into the same family, given that no known morphological characters can be used as synapomorphies of such a taxon. The characters considered as autapomorphies of *Levantoniscus* by Cardoso et al. (2015) could as well define a separate new family. A more inclusive phylogeny is required before we can decide on its familial status, given also the lack of robust synapomorphies defining Trachelipodidae, a family in need of a sound revision.

As indicated by the tree topology, Porcellionidae is more closely related to Trachelipodidae and Agnaridae rather than Armadillidiidae. A similar result has been found by Lins et al. (2017), even though these authors had included only two species in two genera (*Porcellio* and *Porcellionides*) of Porcellionidae in their analysis. It is evident that morphological characters traditionally used in Oniscidea systematics, such as the structure of pleopodal lungs, the number of flagellar segments and the head structure, do not seem to provide adequate evidence that support a robust taxonomy, at least not in all cases.

In conclusion, the monophyly of Porcellionidae as currently perceived cannot be supported by molecular evidence. Of course, we still need to identify phenotypic synapomorphies defining the family, since the characters used so far cannot be considered as valid. In addition, the genus *Porcellio* needs to be revised, as it appears to be polyphyletic, comprising of at least two separate groups.

The monophyletic subgroup of Porcellionidae seems to have an African origin, diverging at the end of the Palaeogene (Oligocene) and then differentiating further during the Miocene. Based on the cladochronology estimated herein, more basal cladogenetic events, leading to the branching of other related families, happened in the Eocene. This chronology is compatible with the very old (Mesozoic) origin of Oniscidea suggested by Broly et al. (2013, 2015).

## Supplementary materials

Supplementary material 1 Percentage sequence divergence among the main clades of Porcellionidae and Maximum Likelihood phylogenetic tree. Explanation note: Each genus comprised a different group, except for Porcellio whose species were treated as different groups because they don't form a monophyletic group. This is an open access article distributed under the terms of the Creative Commons Attribution License (CC BY 4.0), which permits unrestricted use, distribution, and reproduction in any medium, provided the original author and source are credited.Dimitriou AC, Taiti S, Schmalfuss H, Sfenthourakis S
