# Supplementary material for: A molecular phylogeny of Porcellionidae (Isopoda, Oniscidea) reveals inconsistencies with present taxonomy
Source: Zookeys. 2018 Dec 3;(801):163–76. doi: 10.3897/zookeys.801.23566 (PMC6300696; doi:10.3897/zookeys.801.23566)
Supplement: Supplementary material 1 — Percentage sequence divergence among the main clades of Porcellionidae and Maximum Likelihood phylogenetic tree. [file zookeys-801-163-s001.docx]

**Supplementary Material**

**Table S1.** Percentage sequence divergence (p-distance) among the main clades of Porcellionidae for 16s. Each genus comprised a different group, except for *Porcellio* whose species were treated as different groups because they don't form a monophyletic group.

| **Group** | **1** | **2** | **3** | **4** | **5** | **6** | **7** | **8** | **9** | **10** | **11** | **12** | **13** | **14** | **15** | **16** |
| --- | --- | --- | --- | --- | --- | --- | --- | --- | --- | --- | --- | --- | --- | --- | --- | --- |
| 1. *Acaeroplastes* |  |  |  |  |  |  |  |  |  |  |  |  |  |  |  |  |
| 1. *Agabiformius* | 22.3 |  |  |  |  |  |  |  |  |  |  |  |  |  |  |  |
| 1. *Leptotrichus* | 21.2 | 22.6 |  |  |  |  |  |  |  |  |  |  |  |  |  |  |
| 1. *Mica* | 23.9 | 21.7 | 22.5 |  |  |  |  |  |  |  |  |  |  |  |  |  |
| 1. *Porcellionides* | 23.4 | 27.3 | 21.8 | 24.7 |  |  |  |  |  |  |  |  |  |  |  |  |
| 1. *Porcellio laevis* | 21.5 | 18.7 | 21.7 | 17.1 | 23.8 |  |  |  |  |  |  |  |  |  |  |  |
| 1. *Porcellio nasutus* | 25.1 | 23.1 | 26.4 | 23.4 | 23.6 | 20.6 |  |  |  |  |  |  |  |  |  |  |
| 1. *Proporcellio* | 21.1 | 23.9 | 21.9 | 24.1 | 18.5 | 21.1 | 23.0 |  |  |  |  |  |  |  |  |  |
| 1. *Soteriscus* | 20.8 | 22.1 | 16.9 | 21.2 | 23.2 | 21.4 | 25.1 | 23.5 |  |  |  |  |  |  |  |  |
| 1. *Thermocellio* | 25.9 | 22.8 | 24.5 | 23.5 | 27.7 | 23.3 | 27.1 | 25.0 | 25.4 |  |  |  |  |  |  |  |
| 1. *Tura* | 29.6 | 30.1 | 33.5 | 29.8 | 31.6 | 28.9 | 36.5 | 28.3 | 31.9 | 31.3 |  |  |  |  |  |  |
| 1. *Uramba* | 29.2 | 26.3 | 27.1 | 25.9 | 28.2 | 25.2 | 29.7 | 28.8 | 27.0 | 19.9 | 31.1 |  |  |  |  |  |
| 1. *Armadillidium* | 20.7 | 24.3 | 21.0 | 25.0 | 26.5 | 22.6 | 25.2 | 21.6 | 22.5 | 23.4 | 29.6 | 26.6 |  |  |  |  |
| 1. *Hemilepistus* | 21.8 | 23.0 | 20.3 | 24.7 | 22.6 | 21.6 | 23.2 | 20.6 | 20.9 | 26.6 | 31.2 | 26.2 | 22.3 |  |  |  |
| 1. *Trachelipus* | 25.2 | 26.5 | 23.1 | 23.4 | 28.2 | 25.0 | 27.2 | 22.4 | 23.0 | 28.8 | 33.0 | 29.3 | 23.0 | 23.0 |  |  |
| 1. *Chaetophiloscia* | 30.1 | 30.5 | 32.1 | 30.3 | 33.6 | 30.7 | 32.5 | 34.6 | 29.3 | 34.3 | 35.3 | 34.6 | 26.4 | 31.5 | 32.4 |  |
| 1. *Actaecia* | 32.8 | 32.9 | 32.4 | 29.3 | 29.6 | 30.1 | 33.2 | 29.9 | 31.5 | 32.5 | 37.6 | 35.0 | 35.6 | 30.8 | 31.5 | 37.9 |

**Table S2.** Percentage sequence divergence (p-distance) among the main clades of Porcellionidae for COI. Each genus comprised a different group, except for *Porcellio* whose species were treated as different groups because they don't form a monophyletic group.

| **Group** | **1** | **2** | **3** | **4** | **5** | **6** | **7** | **8** | **9** | **10** | **11** | **12** | **13** |
| --- | --- | --- | --- | --- | --- | --- | --- | --- | --- | --- | --- | --- | --- |
| 1. *Acaeroplastes* |  |  |  |  |  |  |  |  |  |  |  |  |  |
| 1. *Caeroplastes* | 28.9 |  |  |  |  |  |  |  |  |  |  |  |  |
| 1. *Lucasius* | 44.9 | 42.2 |  |  |  |  |  |  |  |  |  |  |  |
| 1. *Proporcellio* | 23.4 | 17.2 | 41.5 |  |  |  |  |  |  |  |  |  |  |
| 1. *Porcellionides* | 20.1 | 23.6 | 45.6 | 16.9 |  |  |  |  |  |  |  |  |  |
| 1. *Porcellio laevis* | 22.6 | 33.4 | 50.3 | 28.5 | 18.2 |  |  |  |  |  |  |  |  |
| 1. *Porcellio nasutus* | 19.5 | 28.3 | 46.8 | 23.8 | 23.3 | 20.9 |  |  |  |  |  |  |  |
| 1. *Soteriscus* | 24.9 | 19.1 | 39.8 | 17.9 | 21.4 | 29.5 | 26.5 |  |  |  |  |  |  |
| 1. *Tura* | 23.7 | 33.5 | 49.6 | 24.4 | 22.7 | 23.3 | 24.4 | 27.3 |  |  |  |  |  |
| 1. *Armadillidium* | 22.6 | 32.8 | 50.8 | 28.5 | 19.5 | 19.2 | 21.4 | 30.8 | 23 |  |  |  |  |
| 1. *Hemilepistus* | 21.2 | 33.4 | 50.0 | 29 | 18.6 | 19.9 | 21.9 | 30.5 | 23.2 | 18.9 |  |  |  |
| 1. *Trachelipus* | 21.1 | 28.0 | 47.0 | 21.4 | 19.9 | 20.8 | 20.1 | 24.8 | 23.8 | 19.2 | 19.3 |  |  |
| 1. *Chaetophiloscia* | 21.9 | 38.2 | 51.4 | 32.1 | 21.4 | 20.7 | 21.1 | 33.3 | 23.9 | 20.2 | 19.8 | 21.8 |  |
| 1. *Actaecia* | 21.1 | 26.1 | 47.0 | 24.4 | 22.0 | 22.2 | 22.8 | 24.3 | 24.3 | 21.2 | 20.6 | 20.5 | 19.6 |

**Table S3.** Percentage sequence divergence (p-distance) among the main clades of Porcellionidae for 18s. Each genus comprised a different group, except for *Porcellio* whose species were treated as different groups because they don't form a monophyletic group.

| **Group** | **1** | **2** | **3** | **4** | **5** | **6** | **7** | **8** | **9** | **10** | **11** | **12** | **13** | **13** | **14** | **15** |
| --- | --- | --- | --- | --- | --- | --- | --- | --- | --- | --- | --- | --- | --- | --- | --- | --- |
| 1. *Acaeroplastes* |  |  |  |  |  |  |  |  |  |  |  |  |  |  |  |  |
| 1. *Agabiformius* | 25.4 |  |  |  |  |  |  |  |  |  |  |  |  |  |  |  |
| 1. *Caeroplastes* | 3.6 | 26.0 |  |  |  |  |  |  |  |  |  |  |  |  |  |  |
| 1. *Leptotrichus* | 13.5 | 25.6 | 14.4 |  |  |  |  |  |  |  |  |  |  |  |  |  |
| 1. *Lucasius* | 10.9 | 25.4 | 11.2 | 12.1 |  |  |  |  |  |  |  |  |  |  |  |  |
| 1. *Porcellio laevis* | 9.7 | 3.4 | 5.4 | 7.5 | 2.4 |  |  |  |  |  |  |  |  |  |  |  |
| 1. *Porcellio nasutus* | 3.6 | 28.5 | 4.1 | 14.6 | 11.2 | 7.5 |  |  |  |  |  |  |  |  |  |  |
| 1. *Soteriscus* | 11.9 | 25.3 | 11.4 | 11.7 | 3.9 | 2.5 | 11.8 |  |  |  |  |  |  |  |  |  |
| 1. *Tura* | 13.9 | 22.6 | 14.9 | 11.6 | 14.5 | 5.6 | 17.0 | 13.0 |  |  |  |  |  |  |  |  |
| 1. *Agnara* | 14.4 | 26.8 | 14.8 | 14.1 | 13.6 | 3.7 | 15.3 | 12.8 | 13.9 |  |  |  |  |  |  |  |
| 1. *Hemilepistus* | 16.0 | 25.2 | 16.2 | 13.4 | 14.7 | 5.5 | 16.9 | 14.7 | 15.0 | 8.2 |  |  |  |  |  |  |
| 1. *Armadillidium* | 11.6 | 25.0 | 12.5 | 10.8 | 9.5 | 8.5 | 11.8 | 9.8 | 11.3 | 12.9 | 12.1 |  |  |  |  |  |
| 1. *Schizidium* | 12.9 | 25.4 | 13.9 | 10.5 | 10.1 | 8.5 | 13.3 | 10.4 | 11.0 | 13.6 | 12.6 | 2.0 |  |  |  |  |
| 1. *Levantoniscus* | 7.1 | 22.3 | 7.4 | 8.4 | 5.9 | 0.9 | 7.6 | 7.4 | 8.6 | 8.9 | 8.8 | 7.1 | 6.9 |  |  |  |
| 1. *Trachelipus* | 17.4 | 31.0 | 17.0 | 13.0 | 16.6 | 12.7 | 19.8 | 16.4 | 19.2 | 15.6 | 16.8 | 11.8 | 10.9 | 12.0 |  |  |
| 1. *Chaetophiloscia* | 12.7 | 24.7 | 13.8 | 9.6 | 10.3 | 8.5 | 13.4 | 11.3 | 10.6 | 13.0 | 12.2 | 4.8 | 3.7 | 5.9 | 10.4 |  |
| 1. *Actaecia* | 18.2 | 28.5 | 19.0 | 13.9 | 16.4 | 8.5 | 21.6 | 16.7 | 18.5 | 16.3 | 16.4 | 7.9 | 9.7 | 9.5 | 19.1 | 8.0 |

**Table S4.** Percentage sequence divergence (p-distance) among the main clades of Porcellionidae for 28s. Each genus comprised a different group, except for *Porcellio* whose species were treated as different groups because they don't form a monophyletic group.

| **Group** | **1** | **2** | **3** | **4** | **5** | **6** | **7** | **8** | **9** | **10** | **11** | **12** | **13** | **14** | **15** | **16** | **17** | **18** | **19** | **20** | **21** |
| --- | --- | --- | --- | --- | --- | --- | --- | --- | --- | --- | --- | --- | --- | --- | --- | --- | --- | --- | --- | --- | --- |
| 1. *Acaeroplastes* |  |  |  |  |  |  |  |  |  |  |  |  |  |  |  |  |  |  |  |  |  |
| 1. *Agabiformius* | 28.9 |  |  |  |  |  |  |  |  |  |  |  |  |  |  |  |  |  |  |  |  |
| 1. *Brevurus* | 32.3 | 34.1 |  |  |  |  |  |  |  |  |  |  |  |  |  |  |  |  |  |  |  |
| 1. *Caeroplastes* | 14.0 | 30.0 | 29.7 |  |  |  |  |  |  |  |  |  |  |  |  |  |  |  |  |  |  |
| 1. *Leptotrichus* | 33.8 | 41.5 | 36.7 | 30.1 |  |  |  |  |  |  |  |  |  |  |  |  |  |  |  |  |  |
| 1. *Lucasius* | 20.4 | 20.6 | 29.5 | 28.2 | 30.1 |  |  |  |  |  |  |  |  |  |  |  |  |  |  |  |  |
| 1. *Mica* | 20.6 | 21.3 | 31.0 | 28.1 | 29.5 | 17.5 |  |  |  |  |  |  |  |  |  |  |  |  |  |  |  |
| 1. *Porcellionides* | 22.2 | 24.0 | 23.4 | 26.4 | 32.2 | 24.6 | 25.5 |  |  |  |  |  |  |  |  |  |  |  |  |  |  |
| 1. *Porcellio laevis* | 20.1 | 20.7 | 30.5 | 26.3 | 28.6 | 19.1 | 17.6 | 26.0 |  |  |  |  |  |  |  |  |  |  |  |  |  |
| 1. *Porcellio nasutus* | 17.7 | 35.9 | 35.8 | 17.2 | 41.0 | 24.1 | 22.9 | 24.4 | 19.9 |  |  |  |  |  |  |  |  |  |  |  |  |
| 1. *Proporcellio* | 12.9 | 17.0 | 20.3 | 17.4 | 25.3 | 20.5 | 20.5 | 19.6 | 20.4 | 17.0 |  |  |  |  |  |  |  |  |  |  |  |
| 1. *Soteriscus* | 22.8 | 21.0 | 32.2 | 30.2 | 30.9 | 16.9 | 17.3 | 25.5 | 22.8 | 26.7 | 20.6 |  |  |  |  |  |  |  |  |  |  |
| 1. *Thermocellio* | 22.0 | 16.7 | 44.2 | 21.4 | 30.4 | 21.5 | 17.9 | 26.9 | 0.4 | 23.5 | 19.8 | 28.4 |  |  |  |  |  |  |  |  |  |
| 1. *Tura* | 31.8 | 29.1 | 33.0 | 30.6 | 36.9 | 17.6 | 21.3 | 24.3 | 20.1 | 34.9 | 19.5 | 20.7 | 16.9 |  |  |  |  |  |  |  |  |
| 1. *Uramba* | 32.8 | 32.9 | 34.8 | 31.6 | 41.2 | 18.9 | 22.0 | 26.0 | 21.5 | 38.8 | 19.5 | 21.8 | 18.3 | 17.2 |  |  |  |  |  |  |  |
| 1. *Armadillidium* | 33.0 | 36.8 | 35.6 | 36.4 | 38.9 | 30.4 | 35.2 | 28.4 | 35.4 | 34.6 | 25.6 | 34.9 | 50.0 | 33.6 | 35.3 |  |  |  |  |  |  |
| 1. *Schizidium* | 31.8 | 34.5 | 34.6 | 27.2 | 37.2 | 25.7 | 27.5 | 25.8 | 26.5 | 33.1 | 20.7 | 28.0 | 25.9 | 30.6 | 33.9 | 25.4 |  |  |  |  |  |
| 1. *Agnara* | 23.6 | 24.5 | 31.1 | 24.6 | 31.3 | 25.0 | 27.4 | 26.1 | 27.1 | 26.4 | 20.2 | 27.7 | 28.5 | 23.3 | 27.2 | 34.3 | 29.2 |  |  |  |  |
| 1. *Hemilepistus* | 33.1 | 41.5 | 38.6 | 28.2 | 42.1 | 26.2 | 25.4 | 27.2 | 23.5 | 38.6 | 20.5 | 27.5 | 26.5 | 35.6 | 38.1 | 36.7 | 36.5 | 22.4 |  |  |  |
| 1. *Levantoniscus* | 24.5 | 29.1 | 30.3 | 25.1 | 33.1 | 23.0 | 23.2 | 23.4 | 22.8 | 28.2 | 17.5 | 25.5 | 25.6 | 26.1 | 28.9 | 30.8 | 28.0 | 25.4 | 31.2 |  |  |
| 1. *Chaetophiloscia* | 27.5 | 32.9 | 35.1 | 24.7 | 32.9 | 22.8 | 22.4 | 25.7 | 21.5 | 31.2 | 21.3 | 23.9 | 19.5 | 32.7 | 33.3 | 31.5 | 24.7 | 23.6 | 35.2 | 29.6 |  |
| 1. *Actaecia* | 33.8 | 38.3 | 39.0 | 36.9 | 40.7 | 28.1 | 28.2 | 29.7 | 29.8 | 38.8 | 23.5 | 32.8 | 46.4 | 39.9 | 42.4 | 31.5 | 32.7 | 30.3 | 41.1 | 34.3 | 33.6 |

**Table S5.** Percentage sequence divergence (p-distance) among the main clades of Porcellionidae for NAK. Each genus comprised a different group, except for *Porcellio* whose species were treated as different groups because they don't form a monophyletic group.

| **Group** | **1** | **2** | **3** | **4** | **5** | **6** | **7** | **8** | **9** | **10** | **11** | **12** | **13** | **14** | **15** | **16** | **17** |
| --- | --- | --- | --- | --- | --- | --- | --- | --- | --- | --- | --- | --- | --- | --- | --- | --- | --- |
| **1.** *Acaeroplastes* |  |  |  |  |  |  |  |  |  |  |  |  |  |  |  |  |  |
| **2.** *Agabiformius* | 7.4 |  |  |  |  |  |  |  |  |  |  |  |  |  |  |  |  |
| **3.** *Brevurus* | 6.6 | 7.0 |  |  |  |  |  |  |  |  |  |  |  |  |  |  |  |
| **4.** *Leptotrichus* | 6.6 | 6.6 | 6.0 |  |  |  |  |  |  |  |  |  |  |  |  |  |  |
| **5.** *Lucasius* | 5.8 | 6.2 | 6.2 | 5.1 |  |  |  |  |  |  |  |  |  |  |  |  |  |
| **6.** *Porcellionides* | 5.1 | 6.0 | 5.9 | 6.0 | 5.1 |  |  |  |  |  |  |  |  |  |  |  |  |
| **7.** *Porcellio* *nasutus* | 3.4 | 7.2 | 7.2 | 6.4 | 4.8 | 3.8 |  |  |  |  |  |  |  |  |  |  |  |
| **8.** *Porcellio* *laevis* | 5.1 | 6.0 | 6.4 | 5.5 | 4.3 | 4.9 | 4.5 |  |  |  |  |  |  |  |  |  |  |
| **9.** *Proporcellio* | 4.5 | 6.2 | 6.6 | 5.7 | 4.7 | 2.6 | 3.4 | 4.5 |  |  |  |  |  |  |  |  |  |
| **10.** *Soteriscus* | 6.0 | 7.7 | 6.0 | 6.6 | 5.2 | 5.1 | 5.6 | 4.6 | 5.5 |  |  |  |  |  |  |  |  |
| **11.** *Tura* | 7.3 | 6.6 | 6.8 | 7.8 | 7.1 | 6.2 | 7.7 | 7.2 | 7.2 | 6.2 |  |  |  |  |  |  |  |
| **12.** *Uramba* | 7.0 | 6.1 | 9.1 | 8.1 | 6.9 | 7.0 | 7.2 | 6.9 | 6.9 | 8.3 | 4.8 |  |  |  |  |  |  |
| **13**. *Agnara* | 7.1 | 5.6 | n/c | 3.0 | 5.1 | 4.6 | 3.9 | 6.7 | 4.4 | 5.9 | 4.9 | 5.1 |  |  |  |  |  |
| **14.** *Hemilepistus* | 6.2 | 7.1 | 4.9 | 6.8 | 5.8 | 4.6 | 4.9 | 6.3 | 5.0 | 4.8 | 5.7 | 7.8 | 3.4 |  |  |  |  |
| **15.** *Levantoniscus* | 8.1 | 8.2 | 8.7 | 8.3 | 7.3 | 7.1 | 7.0 | 7.1 | 6.7 | 8.5 | 9.4 | 9.0 | 6.5 | 7.5 |  |  |  |
| **16.** *Trachelipus* | 6.5 | 7.1 | 5.9 | 5.7 | 5.3 | 4.4 | 4.4 | 6.6 | 4.5 | 4.8 | 6.6 | 7.8 | 2.0 | 3.6 | 6.5 |  |  |
| **17.** *Chaetophiloscia* | 11.1 | 10.9 | 10.0 | 10.0 | 7.9 | 10.3 | 9.4 | 10.7 | 9.1 | 10.5 | 11.1 | 12.2 | 8.9 | 9.4 | 11.4 | 8.7 |  |
| **18.***Actaecia* | 20.6 | 18.3 | 22.7 | 20.4 | 18.0 | 19.2 | 19.3 | 19.2 | 18.6 | 19.4 | 18.8 | 20.3 | 12.3 | 16.4 | 20.3 | 18.7 | 17.3 |


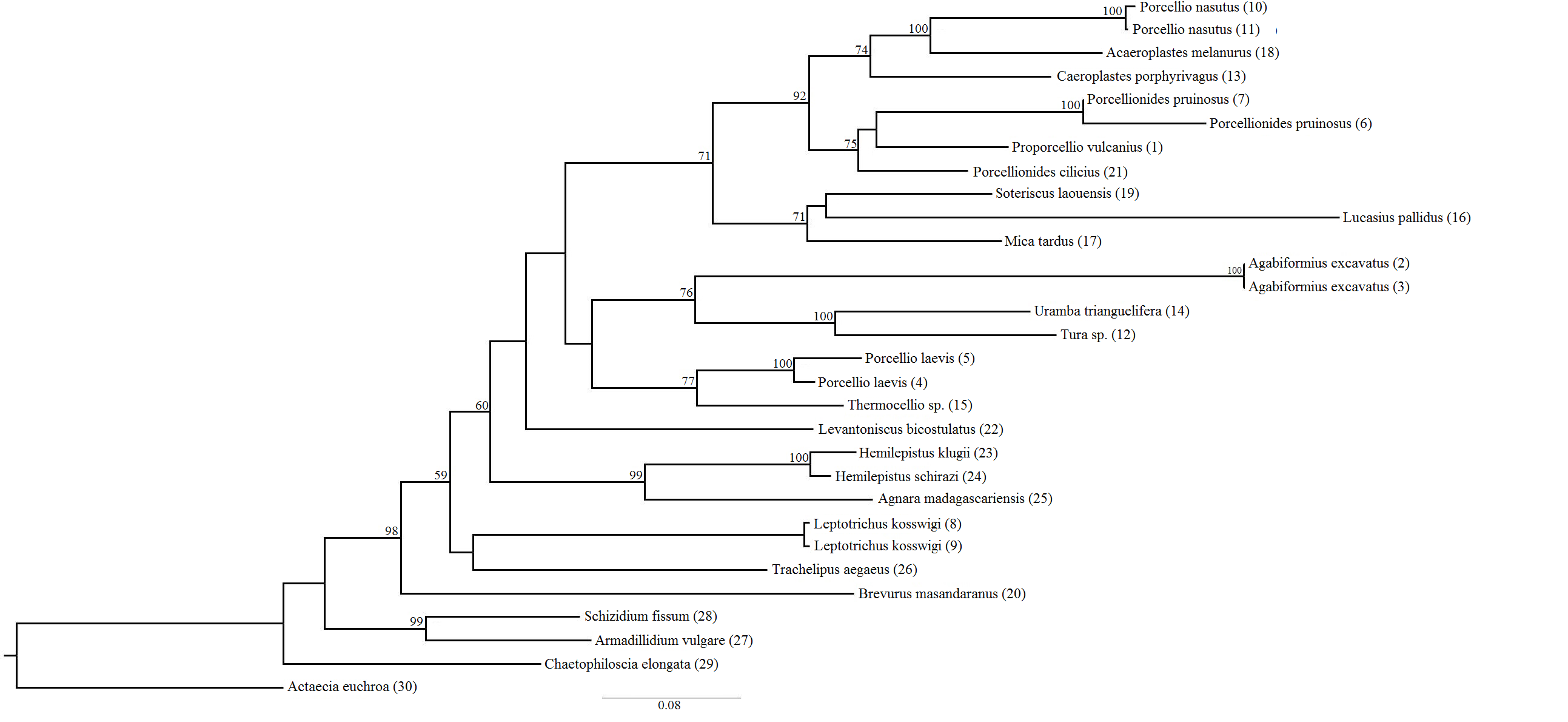
Figure S1: Maximum likelihood phylogenetic tree constructed using 5 genes (COI, 16s, 18s, 28s,NAK). Only the bootrrap values above 50 are given on the nodes.
